# Supplementary figures and images for: On the difficult evolutionary transition from the free-living lifestyle to obligate symbiosis
Source: PLoS One. 2020 Jul 30;15(7):e0235811. doi: 10.1371/journal.pone.0235811 (PMC7392539; doi:10.1371/journal.pone.0235811)

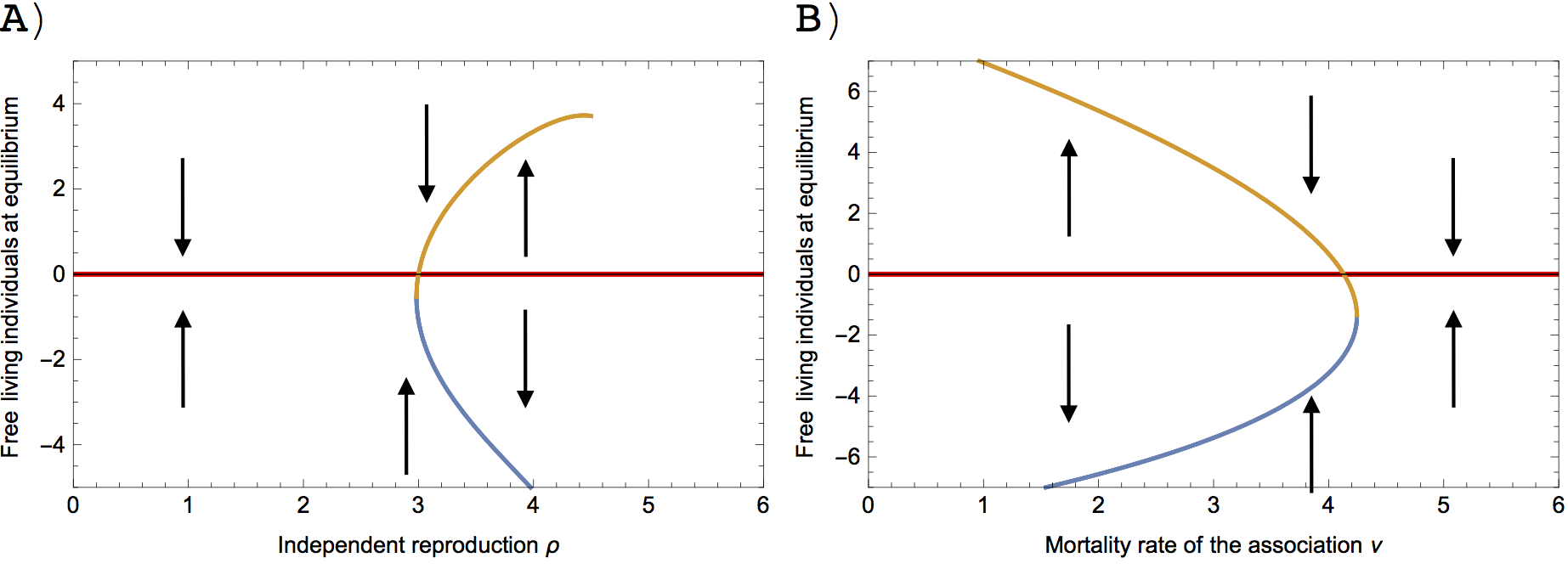

Supplement: S1 Fig — (TIF) [file pone.0235811.s001.tif]
