# Supplementary material for: On the difficult evolutionary transition from the free-living lifestyle to obligate symbiosis
Source: PLoS One. 2020 Jul 30;15(7):e0235811. doi: 10.1371/journal.pone.0235811 (PMC7392539; doi:10.1371/journal.pone.0235811)
Supplement: S1 Appendix — (PDF) [file pone.0235811.s002.pdf]

S1 Stability of the resident population equilibrium

The equilibrium of the resident population can be calculated by solving

$$\begin{cases} \rho\mathcal{F} + \tau\mathcal{A} - \beta(N-\mathcal{A})\mathcal{F} - \mu_0(1+c\mathcal{F})\mathcal{F} = 0 \\ \\ \beta(N-\mathcal{A})F + \sigma\mathcal{A} - \nu\mathcal{A} = 0 \end{cases}$$

which results in 3 equilibria, one of which is the trivial equilibrium $\hat{\mathcal{F}} = \hat{\mathcal{A}} = 0$. The other two non-zero equilibria can be expressed as

$$\begin{cases} \hat{\mathcal{F}}_1 = -\dfrac{X + \sqrt{X^2 - 4c\beta\mu_0 Y}}{2c\beta\mu_0} \quad ; \quad \hat{\mathcal{A}}_1 = \dfrac{Y + (\nu-\sigma)c\mu_0\hat{\mathcal{F}}_1}{\beta(\nu-\tau-\ \sigma)} & \text{(A1)} \\ \hat{\mathcal{F}}_2 = -\dfrac{X - \sqrt{X^2 - 4c\beta\mu_0 Y}}{2c\beta\mu_0} \quad ; \quad \hat{\mathcal{A}}_2 = \dfrac{Y + (\nu-\sigma)c\mu_0\hat{\mathcal{F}}_2}{\beta(\nu-\tau-\ \sigma)} & \text{(A2)} \end{cases}$$

where

$$X = \beta(\mu_0 - \rho) + c\mu_0(\nu - \sigma)$$

$$Y = \beta N(\nu - \tau - \sigma) + (\rho - \mu_0)(\sigma - \nu)$$

We are only interested in positive stable equilibria, which can be either (A1) or (A2) or both. Since their expressions are rather complicated, instead of analysing their stability and sign, we start with analysing the condition in which the trivial equilibrium is unstable.

The trivial equilibrium is unstable when at least one of the eigenvalues of the Jacobian matrix evaluated at $\hat{\mathcal{F}} = \hat{\mathcal{A}} = 0$

$$J = \begin{pmatrix} -\mu_0 - \beta N + \rho & \tau \\ \beta N & -\nu + \sigma \end{pmatrix}$$

is positive. Since the jacobian matrix J is a 2x2 matrix, we can analyse the sign of its eigenvalues based on its determinant and trace. Matrix J has at least one positive eigenvalue when

$$\left[\begin{array}{l} Det(J) < 0 \qquad \text{(A3)} \\ Det(J) > 0 \; and \; Tr(J) > 0 \qquad \text{(A4)} \end{array}\right.$$

Condition (A3) leads to $Det(J) = \beta N(\nu - \tau - \sigma) + (\rho - \mu_0)(\sigma - \nu) = Y < 0$. Condition (A4) results in $-(\nu - \sigma) - \beta N > (\mu_0 - \rho) > -\beta N + \beta N\tau/(\nu - \sigma)$, which is impossible because we assume always that $\nu - \sigma > 0$ to ensure the associated population is within bound, hence, $-(\nu - \sigma) < 0$ and $\beta N\tau/(\nu - \sigma) \geq 0$, which results in $-\beta N - (\nu - \sigma) < -\beta N + \beta N\tau/(\nu - \sigma)$, keeping in mind that all other parameters are positive. Therefore, we need only analyse condition (A3).

Condition (A3) gives

$$|X| - \sqrt{X^2 - 4c\beta\mu_0 Y} < 0.$$

Under such condition, the trivial equilibrium is unstable, which mean that either (A1) or (A2) or both of them are stable. Regardless of the sign of X, the signs of the two non-trivial equilibria are

$$\begin{cases} -\dfrac{X + \sqrt{X^2 - 4c\beta\mu_0 Y}}{2c\beta\mu_0} = \hat{\mathcal{F}}_1 < 0 \\ -\dfrac{X - \sqrt{X^2 - 4c\beta\mu_0 Y}}{2c\beta\mu_0} = \hat{\mathcal{F}}_2 > 0 \end{cases}$$

In all cases, only equilibrium $\hat{\mathcal{F}}_2$ is positive, hence feasible for the mutant invasion analysis. We are aware of the fact that an unstable trivial equilibrium does not necessarily mean

that the positive equilibrium that we use is stable. However, we believe that under our simple ecological dynamics, there is no complication in the stability of the equilibrium. We did not do a thorough analysis because it is not the focus of our study. However, we did a numerical analysis, showing that for small value of independent reproduction ρ and high value of the bound mortality rate ν, the nontrivial equilibrium is stable, whereas, with the reverse conditions, there are two stable equilibria, one of which is always negative. Therefore, there exists only one feasible equilibrium for the invasion analysis (Figure S1).

We observe that the higher the value of ρ, the more negative $Det(J)$ will be. At the extreme case when $\rho = 0$, then $Y = \beta N(\nu - \tau - \sigma) - \mu_0(\sigma - \nu)$. Now, $Y < 0$ when $\beta N(\nu - \tau - \sigma) < \mu_0(\sigma - \nu)$. Since we assume that $\nu > \sigma$, the condition $Y < 0$ is guaranteed only when $\nu - \tau - \sigma < 0$
