# Supplementary material for: On the difficult evolutionary transition from the free-living lifestyle to obligate symbiosis
Source: PLoS One. 2020 Jul 30;15(7):e0235811. doi: 10.1371/journal.pone.0235811 (PMC7392539; doi:10.1371/journal.pone.0235811)
Supplement: S2 Appendix — (PDF) [file pone.0235811.s003.pdf]

S2 Next generation method

We calculate the reproduction ratio R_0 based on the next generation method (Hurford 2010). When mutations are rare, a mutant only occurs when the resident is at its equilibrium $(\hat{\mathcal{F}}, \hat{\mathcal{A}})$. Assuming that the abundance of the new arising mutant is extremely low that thus does not have yet any effect on the resident population density, then the dynamic of the mutant population can be expressed as

$$\dot{\mathcal{F}}_m = \rho\mathcal{F}_m + \tau\mathcal{A}_m - \beta(N - \hat{\mathcal{A}})\mathcal{F}_m - \mu_0(1 + c\hat{\mathcal{F}})\mathcal{F}_m$$

$$\dot{\mathcal{A}}_m = \beta(N - \hat{\mathcal{A}})\mathcal{F}_m + \sigma\mathcal{A}_m - \nu\mathcal{A}_m$$

The differential equations be rewritten as $\dot{\mathbf{u}} = \mathbf{M}\mathbf{u}$, where $\mathbf{u} = \{\mathcal{F}_m, \mathcal{A}_m\}$ and

$$\mathbf{M} = \begin{pmatrix} \rho - \beta(N - \hat{\mathcal{A}}) - \mu_0(1 + c\hat{\mathcal{F}}) & \tau \\ \beta(N - \hat{\mathcal{A}}) & -\nu + \sigma \end{pmatrix}$$

Matrix $\mathbf{M}$ can be partitioned into two components $\mathbf{M} = \mathbf{F} - \mathbf{V}$, where

$$\mathbf{F} = \begin{pmatrix} \rho & \tau \\ 0 & 0 \end{pmatrix}$$

describes the contributions of each state (i.e. the free-living state and the associated state) to the population of the next generation and

$$\mathbf{V} = \begin{pmatrix} \beta(N - \hat{\mathcal{A}}) + \mu_0(1 + c\hat{\mathcal{F}}) & 0 \\ -\beta(N - \hat{\mathcal{A}}) & \nu - \sigma \end{pmatrix}$$

describes the transition rate from one state to the other. According to the next generation theorem, the reproduction ratio of the mutant is the maximum modulus of all the eigenvalues of the matrix $\mathbf{F}\mathbf{V}^{-1}$. This is exactly the eigenvalue of this matrix since it has only one non-zero eigenvalue.
