# Supplementary material for: On the difficult evolutionary transition from the free-living lifestyle to obligate symbiosis
Source: PLoS One. 2020 Jul 30;15(7):e0235811. doi: 10.1371/journal.pone.0235811 (PMC7392539; doi:10.1371/journal.pone.0235811)
Supplement: S3 Appendix — (PDF) [file pone.0235811.s004.pdf]

S3 Conditions for intermediate evolutionarily stable strategy (ESS)

An ESS x^* satisfies

$$\left.\frac{\partial R_0(x,x_r)}{\partial x} = 0\right|_{x=x_r=x^*}$$

$$\iff \left.\frac{(\mathcal{C}_f(x)' + \mathcal{C}_a'(x,x_r))\mathcal{T}(x,x_r)}{\mathcal{T}(x,x_r)^2} - \frac{(\mathcal{C}_f(x) + \mathcal{C}_a(x,x_r))\mathcal{T}'(x,x_r)}{\mathcal{T}(x,x_r)^2} = 0\right|_{x=x_r=x^*}$$

Henceforth, for the sake of brevity,

all the primes indicate the derivative with respect to the mutant trait x

$$\iff \left.\frac{\mathcal{C}_f'(x) + \mathcal{C}_a'(x,x_r)}{\mathcal{T}(x,x_r)} - \frac{\mathcal{T}'(x,x_r)}{\mathcal{T}(x,x_r)} = 0\right|_{x=x_r=x^*}$$

because $R_0(x,x_r)|_{x=x_r=x^*} = \left.\frac{\mathcal{C}_f(x) + \mathcal{C}_a(x,x_r)}{\mathcal{T}(x,x_r)}\right|_{x=x_r=x^*} = 1$

$$\iff \mathcal{C}_f'(x) + \mathcal{C}_a'(x,x_r) = \mathcal{T}'(x,x_r)|_{x=x_r=x^*}$$

$$\iff \left.\rho(x)' + \frac{\hat{\mathcal{H}}}{\mathcal{M}}\left(\beta(x)'\tau(x) + \beta(x)\tau(x)'\right) = \hat{\mathcal{H}}\beta(x)'\right|_{x=x_r=x^*}$$

$$\iff \left.\rho(x)'\left(1 + \frac{\hat{\mathcal{H}}\beta(x)\tau(\rho)'}{\mathcal{M}}\right) = \beta(x)'\left(\hat{\mathcal{H}} - \frac{\hat{\mathcal{H}}}{\mathcal{M}}\tau(x)\right)\right|_{x=x_r=x^*}$$

because $\tau(x)' = \rho(x)'\tau(\rho)'$

$$\iff \left.\frac{\beta(x)'}{\rho(x)'} = \frac{\mathcal{M} + \hat{\mathcal{H}}\beta(x)\tau(\rho)'}{\hat{\mathcal{H}}(\mathcal{M} - \tau(x))}\right|_{x=x_r=x^*}$$

This is exactly expression (10).

An ESS x^* has to be at a maximum of the fitness function, hence, it needs also satisfy

$$\begin{aligned}
&\frac{\partial^2 R_0(x,x_r)}{\partial x_r^2}\bigg|_{x=x_r=x^*} < 0 \\
\iff &\frac{(\mathcal{C}_f''(x)+\mathcal{C}_a''(x,x_r))\mathcal{T}(x,x_r)}{\mathcal{T}(x,x_r)^2} - \frac{(\mathcal{C}_f'(x)+\mathcal{C}_a'(x,x_r))\mathcal{T}'(x,x_r)}{\mathcal{T}(x,x_r)^2} \\
&- \frac{\mathcal{T}''(x,x_r)\mathcal{T}(x,x_r)}{\mathcal{T}(x,x_r)^2} + \frac{\mathcal{T}'(x,x_r)\mathcal{T}'(x,x_r)}{\mathcal{T}(x,x_r)^2} < 0\bigg|_{x=x_r=x^*} \\
\iff &\frac{\mathcal{C}_f''(x)+\mathcal{C}_a''(x,x_r)}{\mathcal{T}(x,x_r)} - \left(\frac{\mathcal{T}'(x,x_r)}{\mathcal{T}(x,x_r)}\right)^2 - \frac{\mathcal{T}''(x,x_r)}{\mathcal{T}(x,x_r)} + \left(\frac{\mathcal{T}'(x,x_r)}{\mathcal{T}(x,x_r)}\right)^2 < 0\bigg|_{x=x_r=x^*}
\end{aligned}$$

because $\mathcal{C}_f'(x)+\mathcal{C}_a'(x,x_r) = \mathcal{T}'(x,x_r)$ at the ESS $x = x_r = x^*$

$$\begin{aligned}
\iff &\mathcal{C}_f''(x)+\mathcal{C}_a''(x,x_r) - \mathcal{T}''(x,x_r) < 0|_{x=x_r=x^*} \\
\iff &\rho(x)'' + \frac{\hat{\mathcal{H}}}{\mathcal{M}}(\beta(x)'\tau(x)+\beta(x)\tau(x)')' - \hat{\mathcal{H}}\beta(x)'' < 0\bigg|_{x=x_r=x^*} \\
\iff &\rho(x)'' + \frac{\hat{\mathcal{H}}}{\mathcal{M}}(\beta(x)''\tau(x)+2\beta(x)'\rho(x)'+\beta(x)\rho(x)'') - \hat{\mathcal{H}}\beta(x)'' < 0\bigg|_{x=x_r=x^*}
\end{aligned}$$

If we assume $\rho(x)' = \tau(x)'$ and $\rho(x)'' = \tau(x)''$

$$\begin{aligned}
\iff &\rho(x)''\left(1+\frac{\hat{\mathcal{H}}\beta(x)}{\mathcal{M}}\right) + \beta(x)''\left(\frac{\hat{\mathcal{H}}\tau(x)}{\mathcal{M}} - \hat{\mathcal{H}}\right) + 2\beta(x)'\rho(x)'\frac{\hat{\mathcal{H}}}{\mathcal{M}} < 0\bigg|_{x=x_r=x^*} \\
\iff &\rho(x)'' + \beta(x)''\frac{\frac{\hat{\mathcal{H}}\tau(x)}{\mathcal{M}} - \hat{\mathcal{H}}}{1+\frac{\hat{\mathcal{H}}\beta(x)}{\mathcal{M}}} + \beta(x)'\frac{2\rho(x)'\frac{\hat{\mathcal{H}}}{\mathcal{M}}}{1+\frac{\hat{\mathcal{H}}\beta(x)}{\mathcal{M}}} < 0\bigg|_{x=x_r=x^*} \\
\iff &\rho(x)'' - \beta(x)''\frac{\rho(x)'}{\beta(x)'} + \frac{\beta(x)'}{\rho(x)'}\frac{2\rho(x)'^2\frac{\hat{\mathcal{H}}}{\mathcal{M}}}{1+\frac{\hat{\mathcal{H}}\beta(x)}{\mathcal{M}}} < 0\bigg|_{x=x_r=x^*} \\
\iff &\rho(x)'' - \beta(x)''\frac{\rho(x)'}{\beta(x)'} + \frac{1+\frac{\hat{\mathcal{H}}\beta(x)}{\mathcal{M}}}{\hat{\mathcal{H}} - \frac{\hat{\mathcal{H}}}{\mathcal{M}}\tau(x)}\frac{2\rho(x)'^2\frac{\hat{\mathcal{H}}}{\mathcal{M}}}{1+\frac{\hat{\mathcal{H}}\beta(x)}{\mathcal{M}}} < 0\bigg|_{x=x_r=x^*} \\
\iff &\rho(x)'' - \beta(x)''\frac{\rho(x)'}{\beta(x)'} + \frac{2\rho(x)'^2}{\mathcal{M} - \tau(x)} < 0\bigg|_{x=x_r=x^*}
\end{aligned}$$

The explicit expression of $\rho(x)$ and $\beta(x)$ suggests that $\rho(x)' > 0$, $\rho(x)'' < 0$, $\beta(x)' < 0$, and $\beta(x)'' > 0$; moreover, at the intermediate x^*, $\mathcal{M} - \tau(x) < 0$. Therefore, $\partial R_0(x, x_r)/\partial x$ will be negative in all cases, implying that if an intermediate singular point exists, it will always be an ESS.
