# Supplementary material for: On the difficult evolutionary transition from the free-living lifestyle to obligate symbiosis
Source: PLoS One. 2020 Jul 30;15(7):e0235811. doi: 10.1371/journal.pone.0235811 (PMC7392539; doi:10.1371/journal.pone.0235811)
Supplement: S4 Appendix — (PDF) [file pone.0235811.s005.pdf]

S4 Effect of the reproduction bonus b on the associated population at equilibrium

The change of the density of the associated population at equilibrium with respect to b is

$$\frac{d\hat{\mathcal{A}}}{db} = \frac{1}{\beta(b-\nu+\rho+\sigma)^2}\Bigg((\mu_0-\rho)(\nu-\sigma) - \beta N(b- \ \nu+\rho+\sigma) + \beta N(-b+\nu-\rho-\sigma) \left(-1+\frac{c\mu_0(\nu-\sigma)}{\sqrt{(\beta(\mu_0-\rho)+c\mu_0(\nu-\sigma))^2 - 4c\beta\mu_0((\mu_0-\rho)(\nu-\sigma)-\beta\ N(b-\nu+\rho+\sigma))}}\right) - \frac{(\nu-\sigma)(\beta(\mu_0-\rho)+c\mu_0(\nu-\sigma))}{2\beta} - \frac{(\nu-\sigma)\sqrt{(\beta(\mu_0-\rho)+c\mu_0(\nu-\sigma))^2 - 4c\beta\mu_0((\mu_0-\rho)(\nu-\sigma)-\beta\ N(b-\nu+\rho+\sigma))}}{2\beta}\Bigg)$$

which can be simplified to

$$\frac{d\hat{\mathcal{A}}}{db} = \frac{\hat{\mathcal{H}} + \frac{c\mu_0\mathcal{M}}{X+2c\hat{\mathcal{F}}\beta\mu_0}N}{\tau-\mathcal{M}}$$

where $\hat{\mathcal{A}} = \mathcal{A}_2$ and $\hat{\mathcal{F}} = \mathcal{F}_2$ as obtained in Appendix S1, $\mathcal{M} = \nu-\sigma$, and $\hat{\mathcal{H}} = N - \hat{\mathcal{A}}$.

At the extreme case when $\rho = 0$, $X = \beta\mu_0 + c\mu_0\mathcal{M} > 0$ and $\tau - \mathcal{M} > 0$ to ensure the stability of the nontrivial equilibrium, hence, $d\hat{\mathcal{A}}/db > 0$. Therefore, the density of the associated population increases with respect to b when $\rho = 0$.
