# Supplementary material for: On the difficult evolutionary transition from the free-living lifestyle to obligate symbiosis
Source: PLoS One. 2020 Jul 30;15(7):e0235811. doi: 10.1371/journal.pone.0235811 (PMC7392539; doi:10.1371/journal.pone.0235811)
Supplement: Attachment — Submitted filename: response_to_reviewer.pdf [file pone.0235811.s008.pdf]

Dear Nick Davies,

Thank you for reviewing our manuscript. You will find below our response to each point that you raised. We hope that they sufficiently clarify your questions.

Abstract: Im confused by the first two sentences. First sentence: Who has suggested this? Endosymbiosis of e.g. mitochondria is often hypothesized to have resulted from a predator-prey scenario, in which case it would not have evolved from what we normally think of as facultative symbiosis. Second sentence: Would e.g. protomitochondria, once engulfed, have the choice of living independently? I feel as though there is one obvious way in which individuals would give up the ability to live freely: the organisms which enter into certain symbioses outcompete those which do not, there are payoffs for closer and closer association (e.g. removal of conflict, better exchange of resources), and over time natural selection acts to remove those stretches of DNA which encode for free living, which are no longer needed and hence are a selective burden. So is this really a riddle? (line 15)

Response: The idea that obligate symbiosis evolves from facultative symbiosis is our extension of Poulin's idea in his book: "Evolutionary Ecology of Parasites" [1]. Here, he suggested that obligate parasites may evolve from facultative parasites if the contact between the parasite and the host is frequent enough [1, Chapter 2]. We argue that the type of ecological interactions may not play a key role in this evolutionary transition, that is, obligate symbiosis may evolve from facultative symbiosis, regardless of whether the interaction is parasitism or mutualism.

We agree that for many symbiotic systems, the associations may have resulted from prey-predator interactions, and if the engulfed symbionts are then maintained purely by vertical transmission with the host then it is

obvious that obligate symbiosis can evolve directly from free-living organisms. However, the prey-predator interaction at the level of microorganisms may completely be different from the interaction at the level of macroorganisms. For instance, a deer is surely dead and cannot escape after being consumed by a tiger, but a bacteria that are engulfed inside a host may escape. In fact, some hydra strains form association with algae by engulfing the algae, but the algae symbionts have been shown to be able to escape from the hydra hosts and enter the external environment ([2]). We argue that the mode of acquiring the symbionts is not that important, and symbiosis formed via engulfing is no different from symbiosis formed via other modes such as rhizobia entering the plant through forming nodules in the roots, or *Vibrio fisherie* entering the bobtail squid in the specialised light organ. Therefore, we find that conventional facultative symbiosis still holds even when it is formed via prey-predator interaction.

In the example that you raised, when protomitochondria were engulfed, it does not follow by being trapped inside their bacterial hosts forever and could only vertically transmit with the hosts and benefit from the hosts. If for any reason, the protomitochondria can escape from the host, they have to deal with the external environment, such as harsh environment, intra and interspecific competition, then they should be better off retaining the ability of independent reproduction, thus, evolving toward obligate symbiosis may not seem so obvious.

Below is our modified paragraph. It can be found in line 22-32 in the revision text file.

" Obligate symbionts have to evolve from free-living individuals, regardless of whether they are parasites or mutualists, because if there were no free-living individuals to begin with, there would be no ingredients for asso-

ciations. If this process occurs due to, for instance, an event of engulfment of the symbiont by the host, followed by purely vertical transmissions of the symbionts to maintain the population then obligate symbionts may evolve directly from free-living organisms. However, many symbionts have to experience external environment before encountering new hosts, and facultative symbiosis is much likely an intermediate step leading toward obligate symbiosis. ”

line 2: contact?

Response: This has been corrected in line 2

27 - 98: This may be a matter of taste, but I feel that a lot of this material going over existing models probably belongs in the discussion. I found it hard to work out from the introduction what the main message of the paper is.

Response: We slightly modified the structure of the introduction, and shorten the explanation of existing models. Below is our modified paragraph. It can be found in line 64-74.

”Theoretical studies that address the evolutionary transition toward obligate symbiosis are rare, but even there, this aspect is not the main focus. Frank [34] only focused on the evolution of cooperation, hence only mutualistic interactions were taken into account. Law & Dieckmann [7] considered parasitism but only evolution of vertical transmission was taken into account; horizontal transmission where symbionts have to experience the external environment was ignored. Van Baalen & Jansen [25], in an analysis of the mutualism-parasitism continuum, pointed out that members of associations are unlikely to give up their evolutionary sovereignty’ as this requires their

interests to be completely aligned, which they claim cannot occur whenever any of the partners has part of its lifecycle outside of the association. However, van Baalen & Jansen [25] did not consider trade-offs that linked traits expressed in the free and the associated states."

39: This paper is just by Frank, not Frank and colleagues.

Response: This has been corrected in line 65.

48: sacrifices

Response: This part has been removed

81: complete

Response: This has been corrected in line 80

100 - 104: There are lots of different kinds of symbiosis in the world and I think it would help to bring in some concrete examples here to clarify what you are modelling. Does this model apply only to endo/ectosymbiosis? Does it also apply to mutualisms like plant-pollinator and cleaner symbioses? I think its more about ecto/endosymbiosis, focusing on the symbiont rather than the host, but because the introduction was quite sweepinge.g. citing in lines 49 previous work that looks very broadly at e.g. mutualisms like cleaner-client and plant-pollinatorits hard to grasp whats being modelled here. Also, the parameter sigma needs to be explained here: when an A makes another A at rate sigma, is the new A associated with the same host as its parent or a new one?

Response: First, we affirm that our model only applies to endo/ectosymbiosis, such as algae-fungi in lichen, aphid-Buchnera, Rhizobia-legume, and so on,

but not to the relationships like plant-pollinator.

Furthermore, we completely agree with the reviewer that the definition of symbiosis is rather ambiguous. When symbioses were discovered in the late 19th centuries, de Bary defined symbiosis as "the living together of unlike named organisms". According to this definition, symbiosis includes both parasitism, mutualism, and associations of all level of partner dependency, from endo/ectosymbiosis, such as lichen, and insects-bacteria to short term symbiosis such as cleaner-client and plant-pollinator. However, it is not very clear whether de Bary did want to include associations such as plant-pollinator or cleaning symbiosis. Zook [3] did mention this ambiguity and redefined symbiosis, that is "Symbiosis is the acquisition of an organism(s) by another unlike organism(s), and through subsequent long-term integration, new structures and metabolism(s) emerge". The symbiotic system that we modelled is closer system defined by Zook [3], that is, it includes only endo/ectosymbiosis.

Furthermore, the parameter σ, vertical transmission is only relevant in the context of endo/ectorsymbiosis. When an association (A) makes another A, the symbiont reproduces with the host, that is the new A is the association of a new symbiont and new host.

It is important to note that in terms of the type of interactions, we still follow de Bary's definition, that is we consider both parasitism and mutualism, even though the type of interactions cannot be taken to account in this model because we considered a fixed number of hosts for simplification.

We also add our affirmation when explaining the model schematic, which can be found in line 100-102.

" It should be noted that we only consider long-term interactions such as lichen, insects and symbiotic bacteria, and so on, instead of the short-term

interactions such as plant-pollinators or cleaning mutualisms. "

105 - 112: So this model cannot analyze vertical transmission? If we are indeed focusing on endo/ectosymbioses, isnt vertical transmission a rather important part of the evolution of obligate symbioses?

Response: We agree with the reviewer that vertical transmission may play an important part in the evolutionary transition toward obligate symbioses and it would be best if we could consider both types of transmissions. However, for various reasons, we focus on horizontal transmission instead of vertical transmission. First, in nature, there are many examples of obligate symbioses with mixed modes of transmission [4]; in fact, many obligate parasites are only horizontally transmitted (it should be noted that we only consider symbionts with simple life cycle, that is symbionts with only one host). Secondly, early in the evolutionary transition from facultative to obligate symbiosis, vertical transmission is likely to be rare as it may require specific adaptations [5]. Therefore, considering the evolution of both types of transmission will make our model much more complicated while we are aiming for a simple model as a start to understand this transition.

117 - 118: Does this assumption reduce the generality of your model? Why was this assumption used to prevent the population of associations from growing without bound, instead of e.g. a saturating growth function?

Response: We do not think that the assumption that the vertical transmission is smaller than the bound mortality rate ($\sigma < \nu$) will reduce the generality of our model because as we responded in the question above, vertical transmission is very likely rare and only happens by chance in the early evolutionary transition. Examples of exclusive vertical transmission

that are observed in nature are mostly systems that are already obligate symbioses, and we cannot be sure whether vertical transmission leads to obligate symbiosis or is it because obligate symbiosis (with horizontal transmission) promotes the evolution of vertical transmission.

The assumption that $\sigma < \nu$ is to prevent the population of A from growing exponentially when the host encounter rate β evolves toward 0. This is the case of completely free-living population because even when there are free-living symbionts and hosts around, the symbionts will not encounter and establish inside the host and form association. If the mutant of zero β is created through horizontal transmission then nothing will happen but if it is created via vertical transmission, then the population of association A will grow exponentially. A saturating growth function require at least quadratic function with regard to the association population, and will prevent analytical results of the equilibrium.

136 - 205: These sections need to be substantially clarified. Moreover, I feel that statements like 167-169 and 190-192, which restate results as statements about geometry, are not helpful. I would appreciate these results being explained in more biological terms. I mostly gave up on the results section at this point.

Response: The entire section of model analysis and result have been modified in line 140-234.

361 - 364: Unclear what this sentence means.

Response: Below are the modified sentences. They can also be found in line 271-280

"The term symbiosis was introduced in the late 1870s after several studies

on lichens had shown that an individual lichen consists of individuals of more than one species [6]. Symbiosis was then considered as a source of evolutionary innovation ([6] and [42] and their references). Symbiosis of prokaryotic cells is suggested to result in eukaryotic cells [3], which is considered one of the major evolutionary transition by Maynard Smith and Szathmary [43], and this is only one extreme case of obligate symbiosis in which obligate dependency is required from both partners. Examples of obligate and facultative symbioses are ubiquitous, with different levels of dependency from both partner. Yet, how obligate symbioses may evolve out of facultative ones is not well-understood, thus studying this intriguing transition process may give insights and guide for future studies. "

409 - 411: The fact that Trichinella can be induced to grow on intestinal epithelial cells in a lab has no bearing on how obligate its symbiosis is in nature. Particularly as the intestine forms part of its natural habitat. What matters is what happens in nature.

Response: Meerovitch et al 1965 successfully cultured *Trichinella Spiralis* in axenic environment. The basal medium used inactivated normal rabbit serum and 25 % chick embryo extract [6].

414: sacrifice

Response: This has been fixed in line 328

Figures, generally: Please provide labels A and B on all the plots, and please left-align, dont right-align these labels. I have never seen this done before and its very confusing. All the plots should have informative axis titles like Fig. 5 so they can more easily stand alone.

Response: The figures have been modified accordingly.

Fig. 2: From the caption and figure alone, its unclear whether this trade-off is a result or an assumption. Obviously this is stated in the text, but it just doesn't help comprehension to have this much ambiguity.

Response: The caption has been modified accordingly.

References

1. Poulin, R. *Evolutionary Ecology of Parasites* 2nd ed. (Princeton University Press, 2006).
2. Miyokawa, R. Y. *et al.* Horizontal transmission of symbiotic green algae between hydra strains. *Arch. Am. Art J.* **235,** 113–122. (2018).
3. Zook, D. in *Reticul. Evol. Symbiogenesis, Lateral Gene Transf. Hybrid. Infect. Hered.* 41–80 (2015).
4. Russell, S. L., Corbett-Detig, R. B. & Cavanaugh, C. M. Mixed transmission modes and dynamic genome evolution in an obligate animal-bacterial symbiosis. *ISME J.* **11,** 1359–1371 (June 2017).
5. Bright, M. & Bulgheresi, S. A complex journey: transmission of microbial symbionts. *Nat. Rev. Microbiol.* **8,** 218–230 (2010).
6. Meerovitch, E. Studies on the in Vitro Axenic Development of *Trichinella Spiralis*. Ii. *Can. J. Zool.* **43,** 81–85 (1965).
