# Supplementary material for: On the difficult evolutionary transition from the free-living lifestyle to obligate symbiosis
Source: PLoS One. 2020 Jul 30;15(7):e0235811. doi: 10.1371/journal.pone.0235811 (PMC7392539; doi:10.1371/journal.pone.0235811)
Supplement: Attachment — Submitted filename: Response_to_reviewers.pdf [file pone.0235811.s009.pdf]

Dear reviewer,

Thank you very much for reviewing our manuscripts. You will find below our response to each point that you raised, and we hope that it will clarify your questions.

The authors mentioned in the supplement that they didn't determine the stability of the positive equilibrium when the trivial equilibrium was unstable. It would be nice add to the supplement some information about the stability of that equilibrium for a few parameter combinations, just as reassurance that nothing strange is going on for some reasonable parameter values.

Response: Indeed, we did not consider the stability of the equilibrium in the first submission. To show that this is not so important in our simple model, and that there is no strange behaviour of the equilibrium, we added in the supplementary document the bifurcation analysis of the equilibrium with respect to the independent reproduction rate ρ and the mortality rate of the association ν. It shows that if the reproduction is not sufficient or if the mortality rate is too high then the trivial equilibrium is stable, whereas in the reverse conditions, there are two stable non zero equilibrium, of which only one is positive and is feasible for the analysis.

Fig. 1 appears to be missing.

Response: We changed the order of the figures when we edited the manuscript and some erroneous references escape our attention. Thank you for pointing this out. We corrected the error.

Lines 82-83: Since symbionts can always reproduce inside the host in this model, it might be better to say that free-living organisms are distinct

from facultative symbionts because they are unable to enter into a symbiotic relationship (beta = 0).

Response: We agree with the reviewer for this clarification and modified the phrase as followed. It is in line 80-83 in the revision manuscript.

"A complete loss of independent reproduction is equivalent to obligate symbiosis, whereas nonzero independent reproduction suggests facultative symbioses with different levels of independent reproduction. Purely free-living organisms do not form associations with the hosts even though they are available."

Lines 109-110: I found this a bit vague. Would it be possible to add that the host population size is kept constant at N?

Response: We added the clarification in line 114-116 in the revision manuscript. Followed is the modified phrase.

"As in this study we aim to focus on the evolutionary pressures on the symbionts, we assume that the total host population size is a constant number N, and we will only consider host dynamics in a future study."

Fig 6 caption: "Black points indicate the ESS values that are corresponded with different values of B." I couldn't find any black points. Does this possibly refer to the colors in the plots?

Response: This is an error that we made during the revision of our manuscript. This phrase should be in the caption of Figure 4, and we corrected this mistake.

Line 254: Is "condition (x)" supposed to have a number in place of x?

Response: This is again an error that we failed to notice. Thank you

for pointing this out. We changed the phrase into

"A consequence of this relaxation is that $\rho'(x) = \tau'(x)$ no longer holds, therefore $d\tau/d\rho$ is no longer a constant, which greatly simplified our analysis."

This is modified in line 255 in the main text.

Lines 263-265: I think this should be "Then, b is no longer a constant but depends on x. As this involves consideration of how the host responds to the symbiont, and thus of coevolution..."

Response: We modified the phrase as followed. We hope that this will clarify what we meant.

"Then, b, the bonus reproduction gained from the exploiting the host, is no longer a constant but depend on x. Moreover, this involves consideration of how the host responds to the symbiont, and thus of coevolution. This falls outside of the scope of this article."

It can be found in line 265-268 in the main text.

Line 301-303: I don't understand this sentence. Does it mean that x will be the same in both cases or that b will become 0?

Response: We modified the paragraph so that our message is clearer. We basically meant that if the symbiont invests the same in independent reproduction and bound reproduction, i.e. $\rho'(x) = \tau'(x)$, evolution toward obligate symbiosis may be more difficult than if it invests differently in the two reproduction rats, i.e. $\rho'(x) \neq \tau'(x)$. In the latter case, the evolution of losing independent reproduction may be favoured depending on the specific assumption of $\rho'(x)$ and $\tau'(x)$.

The following modified paragraph can be found in line 297-306 in the main text.

"This is because we assume that a symbiont invests in its free reproduction the same way that it does in its bound reproduction; what eventually keeps the symbiont in the association is the reproduction bonus that it receives from its host, which is assumed to be a constant amount in the scope of our model.

There may be two reasons that the symbionts invest the same in reproduction regardless of whether it is free-living or associated with its host. First, the symbiont is not sensitive enough to perceive the differences between the external and the host environment. Second, the two environments are so similar that the symbiont does not need to respond differently. For either reason, obligate symbiosis is unlikely to evolve."

Supplement: μ here is μ_0 in the main text.

Response: We thank the reviewer for pointing this out. We changed the mathematical annotations in the supplementery document so that it matches those in the main text.
